# Reply to: “External validation of a simple risk stratification for portal hypertension-related clinical deterioration in patients with ascites”

**DOI:** 10.1016/j.jhepr.2026.101931

**Published:** 2026-06-19

**Authors:** Lorenz Balcar, Marta Tonon, Joan Valls, Thomas Reiberger, Juan-Carlos García-Pagán, Georg Semmler, Salvatore Piano

**Affiliations:** 1 Division of Gastroenterology and Hepatology, Department of Medicine III, Medical University of Vienna, Vienna, Austria; 2 Vienna Hepatic Hemodynamic Lab, Division of Gastroenterology and Hepatology, Department of Medicine III, Medical University of Vienna, Vienna, Austria; 3 Clinical Research Group MOTION, Medical University of Vienna, Vienna, Austria; 4 Unit of Internal Medicine and Hepatology (UIMH), Department of Medicine (DIMED), University of Padova, Italy; 5 Barcelona Clinical Coordinating Center, Barcelona, Spain; 6 Department of Nursing and Physiotherapy, University of Lleida, Lleida, Spain; 7 Biomedical Research Institute of Lleida Fundació Dr. Pifarré (IRBLleida), Lleida, Spain; 8 Barcelona Hepatic Hemodynamic Laboratory, Liver Unit, Hospital Clínic, FRCB-IDIBAPS (Fundació de Recerca Clínic Barcelona-Institut d’Investigacions Biomèdiques August Pi i Sunyer), Barcelona, Spain; 9 CIBEREHD (Centro de Investigación Biomédica en Red. Enfermedades Hepáticas y Digestivas), Health Care Provider of the European Reference Network on Rare Liver Disorders (ERN RARE-Liver), CSUR Centro de referencia del Sistema Nacional de Salud en Enfermedad Hepática Compleja, Departament de Medicina i Ciències de la Salut, Universitat de Barcelona, AGAUR SGR2021 01115, Catalonia, Spain

To the Editor:

We want to thank Gioia *et al.* for providing another external validation and sharing important considerations on the selection of patients with cirrhosis for early transjugular intrahepatic portosystemic shunt (TIPS) placement for ascites.^1^^,^^2^

After Zanetto and Senzolo,^3^ this is the second external validation of our ascites ‘high-risk’ criteria that may serve to identify patients with cirrhosis potentially benefitting from early TIPS placement. By applying the same inclusion and exclusion criteria used in our study, they included 55 patients and found an incidence of clinical deterioration within 12 months of 66.7% among patients with grade 3 ascites and serum sodium ≤135 mmol/L, similar to the incidence rate of 64-69% in our cohort and 61% by Zanetto and Senzolo.^3^

The authors note that – although the ‘high-risk’ criteria include hyponatraemia, a marker of circulatory dysfunction and portal hypertension severity – hepatic encephalopathy (HE) was not considered in our proposed stratification model. Importantly, our study included patients with ascites as the index decompensation event but without overt HE at study inclusion.^4^ In addition, HE was not included in the primary composite endpoint defining clinical deterioration potentially preventable by TIPS.

While the increased risk of HE after TIPS remains a recognised concern, the overall impact of TIPS on HE is likely more complex. By controlling portal hypertension and ascites, TIPS may reduce complications that precipitate HE, including spontaneous bacterial peritonitis, acute kidney injury, severe hyponatraemia, and the need for high-dose diuretic therapy for treating recurrent/refractory ascites. Thus, the risk of HE directly attributable to TIPS should be interpreted in the context of a potentially lower burden of HE due to treatment of portal hypertension. Observations in patients receiving pre-emptive TIPS for variceal bleeding support this, where improved portal hypertension control was not associated with a higher overall incidence of HE.^5^ Moreover, with the first EASL clinical practice guidelines on TIPS in 2025,^6^ management of patients undergoing TIPS has become more standardised, including recommendations for post-TIPS diuretic adjustment. Combined with changes regarding the technical aspects of TIPS placement (such as consideration of smaller diameter stents), these developments have substantially improved care for patients undergoing TIPS and are also expected to reduce the incidence of HE.^7^^,^^8^ We also understand the concerns about potential complications of TIPS and are convinced that decisions regarding early TIPS for ascites should be guided by strong evidence. Strong evidence, however, can only be generated by prospective randomised controlled trials – building on strong rationales, such as risk models that can be consistently validated in external cohorts. This will eventually improve our understanding of the potential benefit of early TIPS placement.^9^^,^^10^

In conclusion, we thank the authors for their external validation of our ascites high-risk criteria that support the concept of earlier TIPS placement in patients with ascites to optimise clinical outcomes.

## Authors' contributions

Drafting of the manuscript (all authors), revision for important intellectual content (all authors).

## Financial support

No financial support was received to produce this manuscript.

## Conflicts of interest

L.B. served as an advisory board member for Boehringer Ingelheim and received travel support from Roche. M.T. received travel support from Gilead and Grifols. J.V. has nothing to disclose. T.R. served as a speaker and/or consultant and/or advisory board member for AbbVie, Bayer, Boehringer Ingelheim, Gilead, Intercept, MSD, Roche, Siemens, and W. L. Gore & Associates and received grants/research support from AbbVie, Boehringer Ingelheim, Gilead, MSD, Philips, and W. L. Gore & Associates as well as travel support from Boehringer Ingelheim and Gilead. J.C.G.-P. received speaker fees from GORE and grants from GORE and Cook. G.S. received speaker fees from DiaSorin. S.P. received fees for consulting/advising from Plasma Protein Therapeutics Association, Boehringer Ingelheim, Mallinckrodt and speaking fees from Grifols, Ferring and Medscape.

Please see the accompanying ICMJE disclosure forms in the Supplementary information for further details.
